# ACTING WITH AWARENESS AND VISUOSPATIAL LEARNING IN MILD COGNITIVE IMPAIRMENT

**DOI:** 10.1093/geroni/igae098.3306

**Published:** 2024-12-31

**Authors:** Melissa Zammitti, Emily Bower, Geoffrey Tremont

**Affiliations:** Pacific University, Portland, Oregon, United States; Pacific University, Hillsboro, Oregon, United States; Brown University, Providence, Rhode Island, United States

## Abstract

Mindfulness practice is a potentially low-risk intervention for mild cognitive impairment (MCI), with numerous studies indicating its robustness in addressing modifiable risk factors associated with the progression of MCI. Evidence suggests a positive association between mindfulness and cognitive ability, including within the visuospatial processing domain; however, which facets of mindfulness are associated with aspects of visuospatial processing are not well understood. We aimed to assess the relationship between the five facets of mindfulness and visuospatial learning and memory (VSLM). We hypothesized that stronger endorsement of the acting with awareness facet would correlate with improved VSLM, as present-mindedness could enhance the processing of one’s physical surroundings. In this secondary analysis, we conducted a hierarchical linear regression using baseline data from a pilot intervention study involving older adults with MCI. 46 participants with amnestic MCI (65% female, mean age 71.6 years) completed the Five Facet Mindfulness Questionnaire (FFMQ) and the Brief Visuospatial Memory Test-Revised (BVMT-R). We controlled for age, race, gender, education, and depression. The analyses revealed a negative relationship between BVMT-R learning scores and FFMQ Acting with Awareness subscale scores (FFMQ-A) such that higher learning was related to lower awareness (β= -.540, p=.021. Associations between BVMT-R scores and other mindfulness facets were not significant (p’s>.05). Findings suggest that the specific domain of mindfulness, acting with awareness, may be associated with poorer visuospatial learning over repeated trials in individuals with MCI. The findings encourage further investigation of the acting with awareness facet and its role in interventions for older adults with MCI.

